# The spatial phase transition of micro/nano particles and its effect on the cleaning efficiency of laser-plasma shock wave cleaning

**DOI:** 10.1038/s41598-023-41405-w

**Published:** 2023-09-04

**Authors:** Shijie Li, Changtao He, Na Xie, Jing Xiao, Junpu Zhao, Jinghua Han, Guoying Feng, Qianqian Song

**Affiliations:** 1 College of Electronics and Information Engineering, Sichuan University, Chengdu, 610064 China; 2 Sichuan Jiuzhou Electric Group Co., Ltd., Mianyang, 621000 China; 3 Laser Fusion Research Center, China Academy of Engineering Physics, Mianyang, 621999 China

**Keywords:** Physics, Optical physics

## Abstract

Plasma cleaning is an effective method for removing micro/nanoparticle particles, thus solving the pollution problem of micro/nanoparticle instruments. However, the lack of research on the phase transition evolution law of micro/nanoparticles under the action of plasma affects the popularization and application of this method and is the key factor that affects the cleaning quality. The focus of this study is to analyze this law. Through experimental observation and finite element simulation, the spatial phase transition distribution characteristics of particles and the influence law of laser parameters are analyzed. Moreover, the effect of the particle phase transition on the cleaning process is discussed. The removal threshold and the best removal area of different particles are presented, and a reference and guidance for the follow-up development of laser-plasma shock wave removal technology are provided.

## Introduction

After more than 20 years of research, laser-plasma cleaning technology has reached a relatively mature stage. Compared with traditional laser cleaning technology, plasma shock wave removal technology has many advantages, such as having high efficiency, providing environmental protection, and causing less damage to the substrate. Since Lee^1^ first solved the problem of tungsten particle removal by laser-plasma shock wave (LSC) in 2001, there has been much corresponding research at home and abroad, among which the research ideas and results are mainly divided into three categories.

One category is the removal mechanism. In 1994, Soltani^2^ proposed three different methods of particle removal, namely, bouncing, sliding, and rolling removals. Later, in 2007, Bian^3^ proposed the removal method of jumping particles. This research is an early study of particle removal and lays a foundation for the subsequent development and research of LSCs.

Second, the characteristics of the spatial shock waves of plasma and its influence on the removal effect are discussed. In 2005, Lim^4^ studied shock waves and obtained the stress of shock waves on particles under different conditions. In 2018, GU^5^ proposed three areas of particle distribution during LSC removal and divided the substrate into three areas, A, B, and C, to discuss the removal effect. In 2021, Zhang et al.^6^ discussed and analyzed the blind area of particle removal. The removal effect and application of LSCs have theoretical support with the distribution law of spatial removal.

The third category is to theoretically and experimentally analyze the thermodynamic effect and phase change characteristics of particles under shock waves and determine the effect of particle removal. Cetinkaya^7^ noted that such particles can be removed at high temperatures due to thermodynamic effects. In 2020, Luo^8^ proposed that particles would melt and break under the action of thermodynamics. In 2021, Lai^9^ also studied the damage of particles to the base and proposed that particles would increase the damage to the base. This is the research trend of this technology and the focus of today's research. This problem provides support for the application and condition control of this technology. Previous results have explored and summarized the principle, mechanism, and efficiency of LSCs and proven the feasibility and operability of this method theoretically and experimentally^10–13^. However, the emphasis is on the stress during removal and the effect after removal, while there is little research on particle removal. Although some studies on the phase transition of particles have been performed under thermal actions, these studies are theoretical, and they do not provide a unified explanation for specific particle sizes and the stress position of particles. By fully explaining and studying the force and heat of particles under different conditions, we can determine the phase change process of particles, their removal mechanism, and their removal effects with different sizes and different areas, thus forming a comprehensive interpretation and combination of LSC technology.

In this study, a complete particle removal process can be obtained by decomposing and analyzing the physical and chemical process of particle removal by plasma and verifying the distribution of substrate residues after laser removal. Through the removal process of particles with different sizes and different distributions, we can determine the movement of particles more intuitively and at the same time provide a threshold of the particle-phase change evolution, crushing, and melting. Moreover, this study provides a reference for removing particles of different sizes and positions.

## Experimental part

### Experimental device

**Figure 1:**
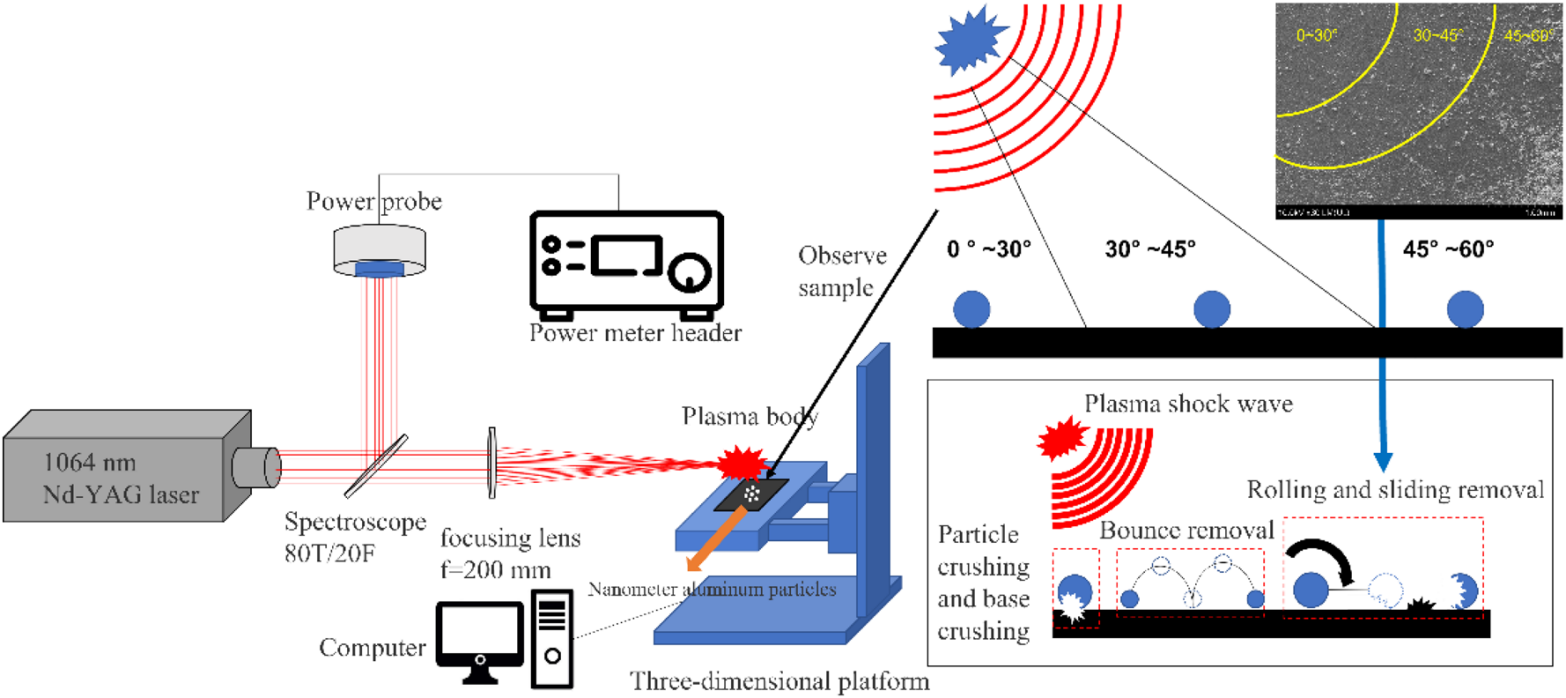
Experimental device diagram.

The experimental device is shown in Fig. 1 above. In the experiment, laser-generated plasma was used to remove Al particles on a Si substrate. The laser used was a Nd:YAG pulse laser with an output wavelength of 1064 nm, pulse width of 12.4 ns, and repetition frequency of 1 Hz. After the pulse laser is output, it passes through a spectroscope (the splitting ratio is 2:8), and the power of the part with less energy is monitored in real time with a power meter. The other part passes through a focusing lens with a focal length of 200 mm to focus on the sample. The sample is placed on a three-dimensional platform, and the computer controls its up and down movement to change the distance between the sample and the plasma.

### Preparation of the experimental samples

The Si sheet was placed in deionized water for ultrasonic cleaning for 30 min, removed, and dried. The 100-nm Al particles were put into ethanol and stirred with a magnetic stirrer for 6 h. The cleaned Si wafer was placed into the prepared Al-ethanol suspension and kept in a dry and ventilated place until the ethanol completely volatilized.

The action sample is placed into the device shown in Fig. 1 for the cleaning experiment and is observed by scanning electron microscopy (SEM). According to the distribution characteristics of particles on the substrate and the action angle of the shock wave on the substrate, the substrate can be approximately divided into three areas, as shown on the right side of Fig. 1.

### Experimental results

According to the angle between the particle position and the plasma explosion point, the whole substrate can be roughly divided into three regions, namely, 0°–30°, 30°–45° and 45°–60°. In addition, the particles in the region of 0°–30° located directly below the plasma explosion point are mainly subject to the vertical force of the shock wave. Particles (30°–45°) located outside the explosion point are subject to the same horizontal and vertical forces of the shock wave. The particles in the region of 45°–60° located at the outermost are mainly subject to the horizontal force of the shock wave.

#### Physical changes of particles

**Figure 2:**
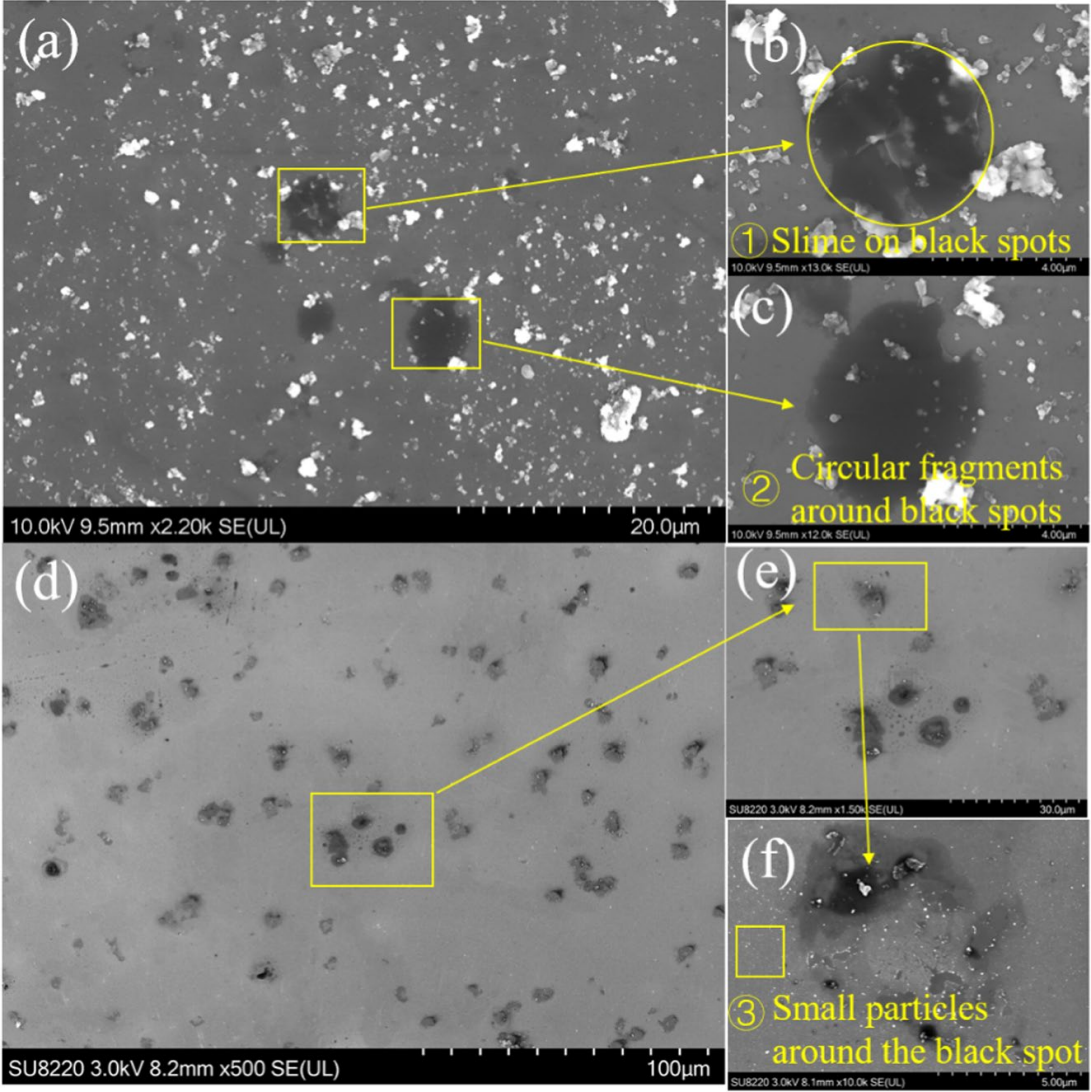
0°–30° SEM image: (**a**) overall image of Black Spot 1; (**b**, **c**) enlarged view of Black Spot 1; (**d**) overall drawing of Black Spot 2; (**e**, **f**) enlarged drawing of Black Spot 2.

Figure 2 presents the result in the range of 0°–30°. Figure 2a and d reveal the presence of many black spots in the range of approximately 500–1000 nm in the region, and many white particles are found in the range approximately between 100 and 300 nm. The SEM images reveal that the conductive part will be darker, while the non-conductive part will be brighter. Therefore, the black spots are aluminum, and the white particles are alumina particles. By enlarging (a), Areas ① and ② of Fig. 2b and c, respectively, reveal that the white particles are distributed in a ring shape around the black spots, and there are signs of melting in the black spots in Area ①. Then, by enlarging the area in Fig. 2d, we can find that there are also black spots and white particles distributed in a ring shape in Fig. 2f, and at the same time, in Area ③, numerous extremely small particles with sizes of approximately 100 nm are distributed. In the range of 0°–30°, the larger particles (500–1000 nm) have almost only one layer of melted broken particles, which are distributed annularly around the original particles, left on the substrate. Smaller particles (100–300 nm) remain on the substrate in large quantities, and no significant change occurs.

**Figure 3:**
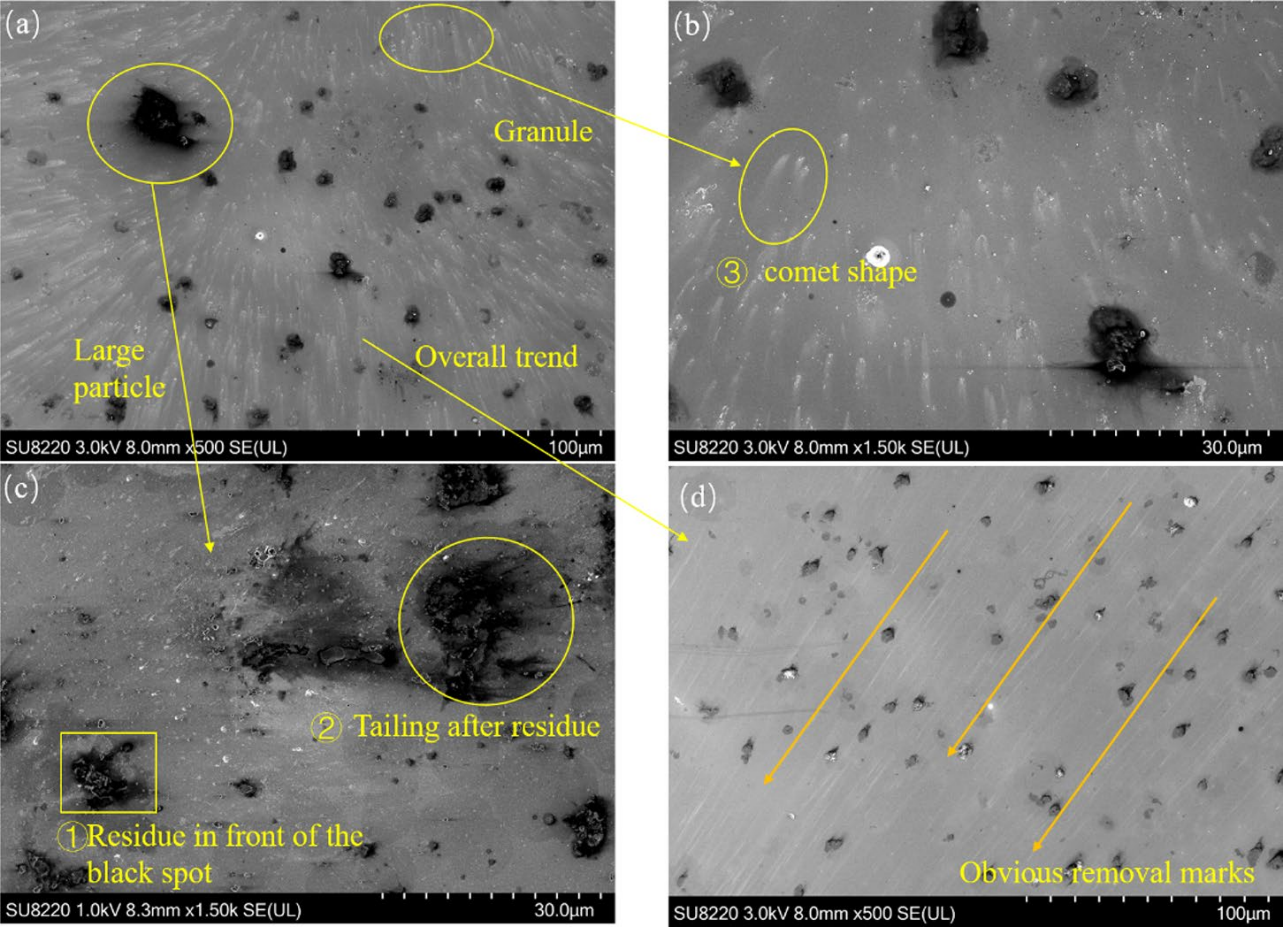
SEM image of the 30° –45° area (**a**) overall view; (**b**) enlarged picture of small particles; (**c**) enlarged view of large particles; (**d**) removal of the trace map.

Regarding the region in the range of 30°–45°, as shown in Fig. 3a, numerous residues and black spots of more than 500 nm occur on the substrate. In contrast, in the range of 0°–30°, the particles around the black spots are not uniformly distributed but have a relatively uniform directional distribution. Through the distribution of the residues, we can see the removal direction of the shock waves. A careful observation of the black spots in Areas ① and ② of Fig. 3c shows that there are still many residues of particles, which are all at the front of the particle removal, which indicates that the large particles will be displaced under the action of the shock wave. However, in the initial state, the particles will be subjected to compressive stress in the vertical direction and a strong friction force in the horizontal direction. Therefore, greater stress exists between the particles and the substrate. The area in Fig. 3b does not contain scratches, but it is composed of a pile of small particles and broken fragments of large particles. In front of these scratches, there is a notable particle gathering place, which forms the head of the comet, followed by scattered particles forming the tail of the comet. They gather with small particles that are not easily destroyed after the large particles are removed and broken, forming the head of the comet. Then, after the strong action of the shock wave, they are dispersed again, and the fragments and small particles are spread out along the direction of the shock wave, forming a tail. Figure 3d reveals a notable particle removal track that is different from the range of 0°–30°, showing the direction of the shock wave.

**Figure 4:**
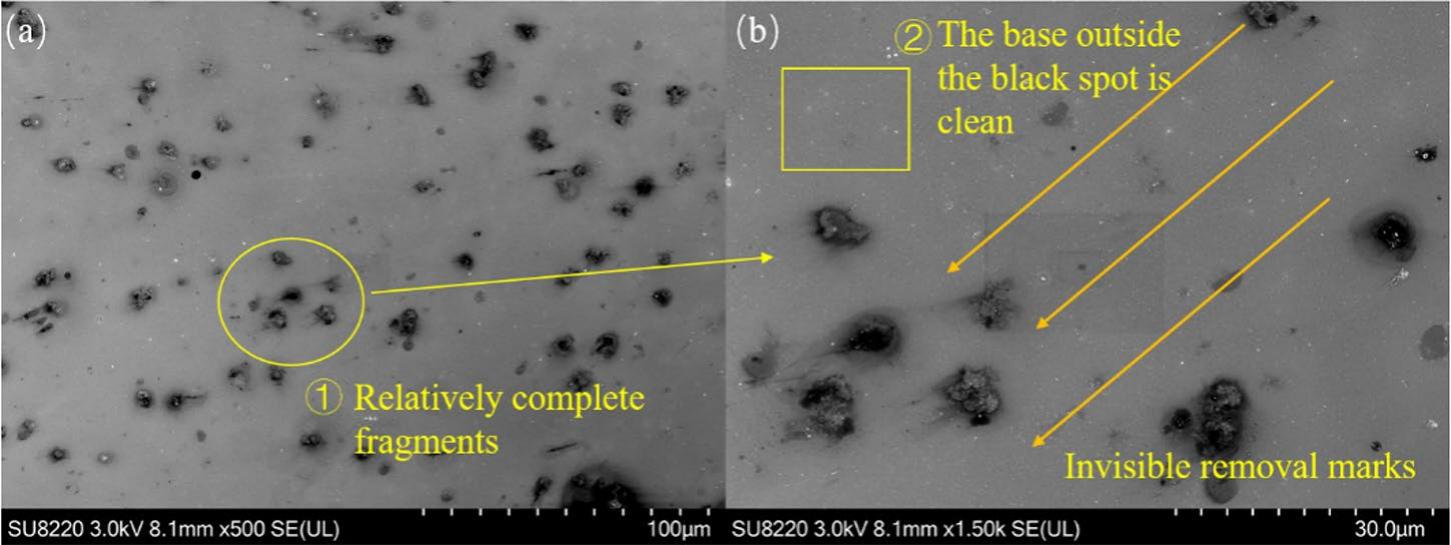
SEM image of the 45°– 60° area (**a**) overall view; (**b**) enlarged drawing.

Figure 4a reveals that in the area in the range of 45°–60°, there are also black spots of micrometer levels but no notable removal marks. In Area ①, particle residues are found at the front end of the removal. By enlarging Fig. 4b, the direction of the shock wave can be determined. However, by comparing Figs. 4a and 3b, the residues occur mostly as entire pieces, without too many tiny fragments. This shows that in this area, the force exerted by the large particles from top to bottom decreases, and the force exerted in the horizontal direction is greater. In the initial state, the static friction force exerted by the substrate on the particles is larger. Therefore, notable breakage occurs at the interface between the particles and the substrate. However, the force exerted in the vertical direction and the stress exerted inside the particles are smaller, and only the bottom of the particles is broken. Because there are not too many broken particles and the clusters of the small particles are not significant, it is impossible to form a comet-like head; thus, there is no notable removal trace on the substrate.

#### Physical changes of the particles

In the previous observation of particle crushing and removal, liquid crystals formed on the surface of the particles. The forming part is just the action area of the shock wave. These crystal juices are formed because of the melting and post-solidification of the particles at high temperature. Therefore, after the shock wave acts on the particles, it not only cause the bottom of the particles to collide with the substrate but also generates significant pressure to the receiving objects. In the process of the laser-plasma shock wave, state changes such as melting and crushing of nanoparticles occur. Through energy-dispersive X-ray spectroscopy (EDS) analysis, we determined that the elements of the nanoparticles before and after the plasma shock wave also have corresponding change rules. Thus, they also reflect different reaction processes.

**Figure 5:**
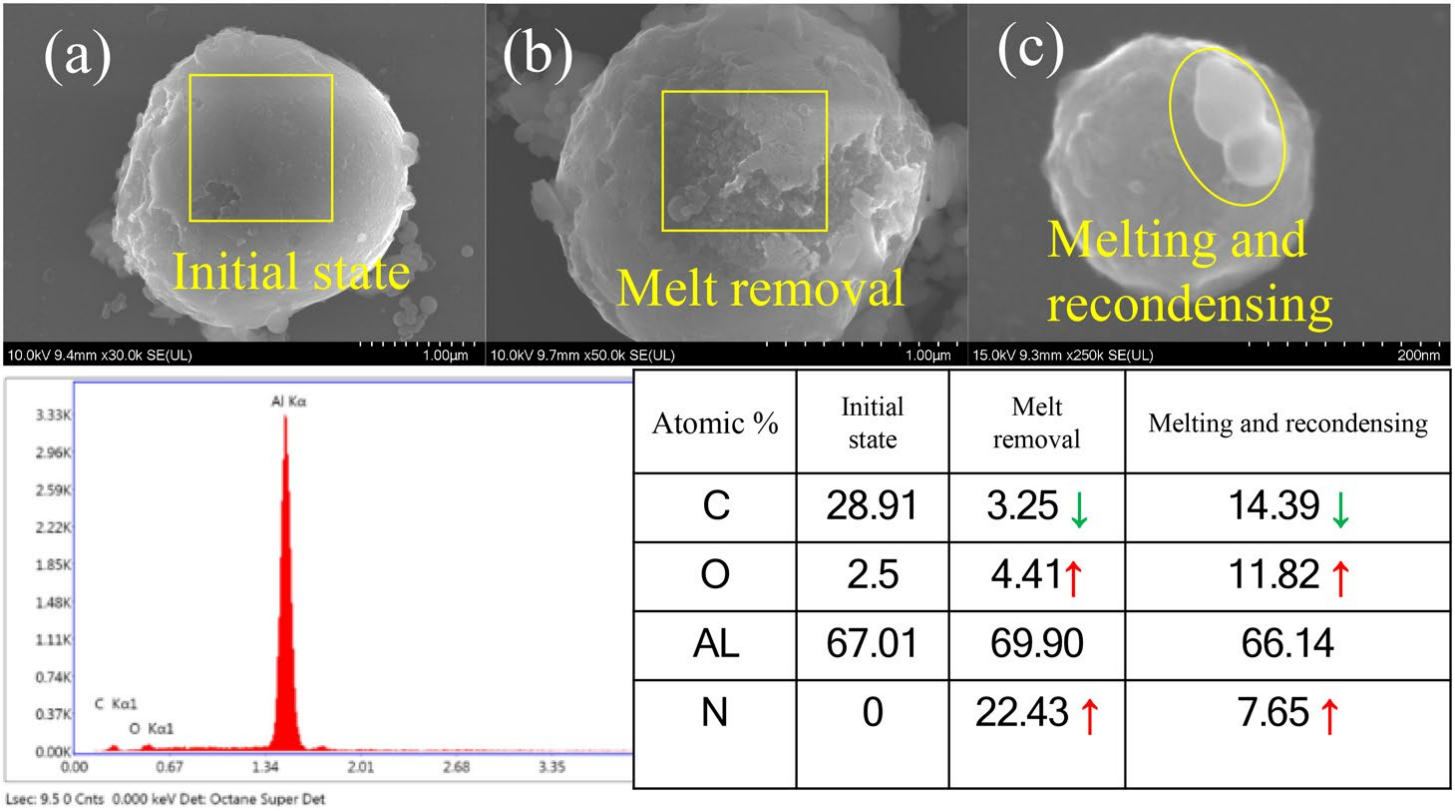
EDS diagram of the particle surface. (**a**) Original sample; (**b**) removal after melting; (**c**) coagulation after melting.

Figure 5a shows the original nanoparticle diagram. According to the element distribution table below, it mainly contains Al, C and O, in which the atomic percentage of C is 28. 91%. Because the SEM penetrates through the air when observing the sample, it causes carbon deposition. Figure 5b and c show that after the plasma shock wave, the nanoparticles are re-coagulated after melting and ablated into cavitation. Figure 5b shows the morphology of the melted nanoparticles, at which time the melted part on the surface of the nanoparticles is removed with a liquid. Figure 5c reveals that the nanoparticles are round and spherical, and some patterns occur at the bottom of the particles. Only when the nanoparticles are melted and condensed can such regular spheres appear. Figure 5b and c show that the C of the nanoparticles decreases, and a new element, N, appears. This is because after C fully interacts with air, CO_2_ is formed and dissipated in the air; thus, C also disappears. N appears because the main linear spectral lines in the laser-plasma spectrum generated by the laser breakdown of air are O and N; therefore, the deposition of N may occur. Regardless of whether the nanoparticles are coagulated after melting or ablated into cavities, the O content increases from the original 2.5% to 4.41% and 11.82%. At this time, the silicon substrate is also ablated, forming silica, and the nanoparticles stick together. Moreover, the content of the ablated elements is inconsistent to different degrees.

## Theoretical analysis

Laser plasma removal mainly depends on the action of the shock waves. As a kind of stress wave, the shock wave propagates inside the particle after acting on the particle, and after propagating to the junction between the particle and the substrate, it is reflected by the substrate and repeatedly propagates inside the particle, causing extrusion and pulling on the particle. Then, due to the high temperature caused by the shock wave, the temperature distribution of the particles is uneven, making the particles easily locally cracked. Moreover, the particles are crushed and melted because of the extrusion of the stress wave. At the same time, the degree of crushing and melting depends on the particle sizes. Therefore, the two main factors that affect the evolution and removal characteristics of micro/nano particles by plasma are the (1) plasma shock waves and (2) particle sizes.

### Parameter setting

**Figure 6:**
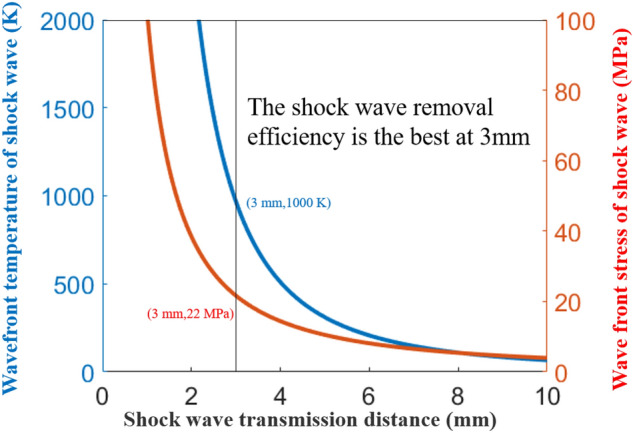
Characteristic diagram of the shock wave.

First, the characteristics of the plasma shock wave include the pressure and temperature on the front of the shock wave. According to the propagation formula and propagation time of the shock wave, the transmission pressure formula of the shock wave can be obtained as follows^14–17^:

1 $$ P = \frac{2}{\gamma + 1}\rho_{0} U^{2} \left( {1 - \frac{\gamma - 1}{{2\gamma }}M^{ - 2} } \right) $$

where γ is the specific heat capacity of air, taken as 4/3; ρ_0_ is the plasma density, which is taken as 1.3; U represents the wave front transmission speed of the shock wave, which is obtained by taking the derivative of the time t through the wave front propagation radius; and M is the maximum Mach number of the initial shock in the instantaneous stage of the shock wave. The red curve in Fig. 6 is obtained according to the formula. When the shock wave travels 3 mm, the pressure of the shock wave front is approximately 22 MPa.

According to the wave front formula of the shock wave^4^, the following equation can be obtained:

2 $$ T = \frac{{2U^{2} \left[ {1 - \frac{\gamma - 1}{{2\gamma }}M^{ - 2} } \right]\left[ {\frac{{\gamma - 1 + 2M^{ - 2} }}{\gamma + 1}} \right]}}{{\left( {\gamma + 1} \right)^{2} }}R_{G} $$

where $R_{G}$ is the universal gas constant. By drawing the blue curve in Fig. 6 using the formula, the temperature of the shock wave is approximately 1000 K when the shock wave reaches 3 mm.

**Figure 7:**
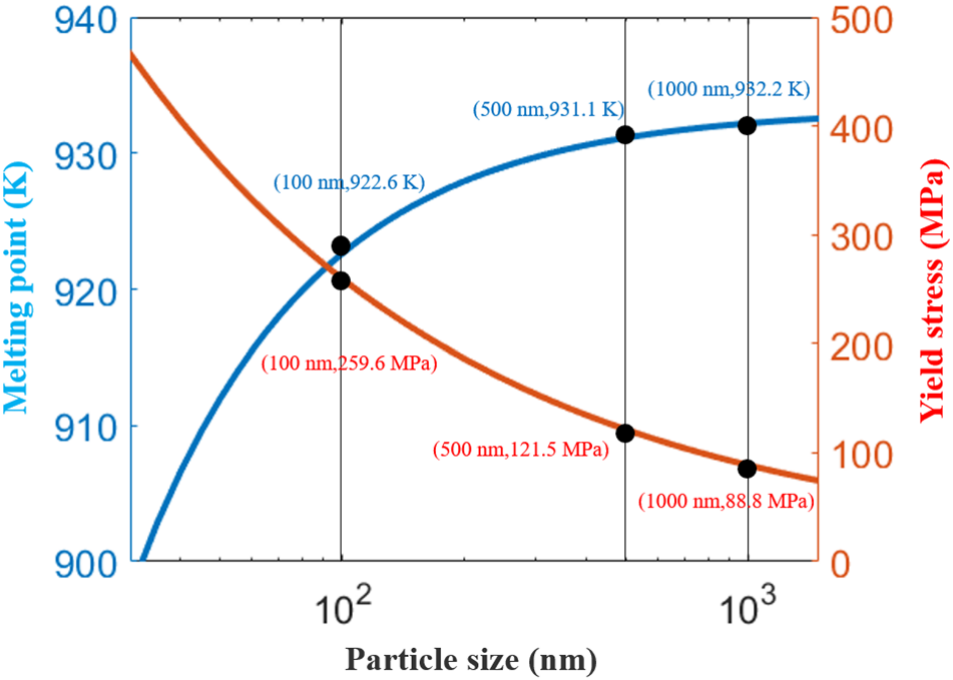
Particle characteristic diagram.

Second, the particle size characteristics, according to the Hall‒Petch formula^17–19^ of the yield stress and particle size, are given as

3 $$ \sigma_{y} = \sigma_{0} + \Delta \sigma_{H - P} $$

4 $$ \Delta \sigma_{H - P} = \frac{{k_{y} }}{\sqrt d } $$

where σ_0_ is the material constant of the initial stress of the dislocation movement (Al = 9.8 MPa), d is the particle diameter, and k_y_ is the strengthening coefficient (k_Al_ = 0.079). According to the formula, the particle size is in the range of 50–1000 nm, and the red curve in Fig. 7 can be obtained. The yield stress of the particles increases with the decrease in the particle diameter. Therefore, when subjected to the same shock wave, large particles are more easily broken after colliding with the substrate, while small particles are removed from the substrate.

The relationship between the particle size and particle melting point is given as^20,21^

5 $$ \frac{{T_{m} }}{{T_{mb} }} = 1 - \frac{\beta }{d} $$

where T_m_ is the melting point of the particles, T_mb_ is the melting point of the corresponding bulk material (T_mb_ of Al = 933.25), β is the material constant (Al_β_ = 1.14), and d is the particle size. Similarly, if the particle size is in the range of 1000–50 nm, the blue curve in Fig. 7 can be obtained. Regarding the melting points in the range of 50–1000 nm, the aluminum particles hardly fluctuate. From 910 to 930 K, the size of the Al particles has little effect on the melting point of the particles.

Next, the phase change of the particles is analyzed by finite element software. The model can be used to build Al particles on a Si substrate. The Si substrate is 50 $\times$ 50 $\times$ 30 µm, and the bottommost surface is set as a fixed point. The sizes of the Al particles are 50 nm, 75 nm, 100 nm, 200 nm, 500 nm, and 1000 nm in diameter. The application of the load exerts a compressive stress of 22 MPa and a temperature load of 1000 K on the upper surface of the particles.

The remaining parameters are given in Table 1^22,23^.

**Table 1:** Related parameters of the Al particles and SI substrate.

Material	Thermal conductivity/pW μm^−1^ K^−1^	Density/kg μm^−3^	Specific heat capacity/pJ kg^−1^ K−^1^	Poisson's ratio	Young’s modulus/MPa	Thermal expansivity
AL	237 × 10^6^	2700 × 10^–18^	880 × 10^12^	0.330	70 × 10^3^	23.21 × 10^–6^
Si	150 × 10^6^	2328 × 10^–18^	618 × 10^12^	0.278	190 × 10^3^	0.50 × 10^–6^

### Influence of the shock wave characteristics on the particle evolution

**Figure 8:**
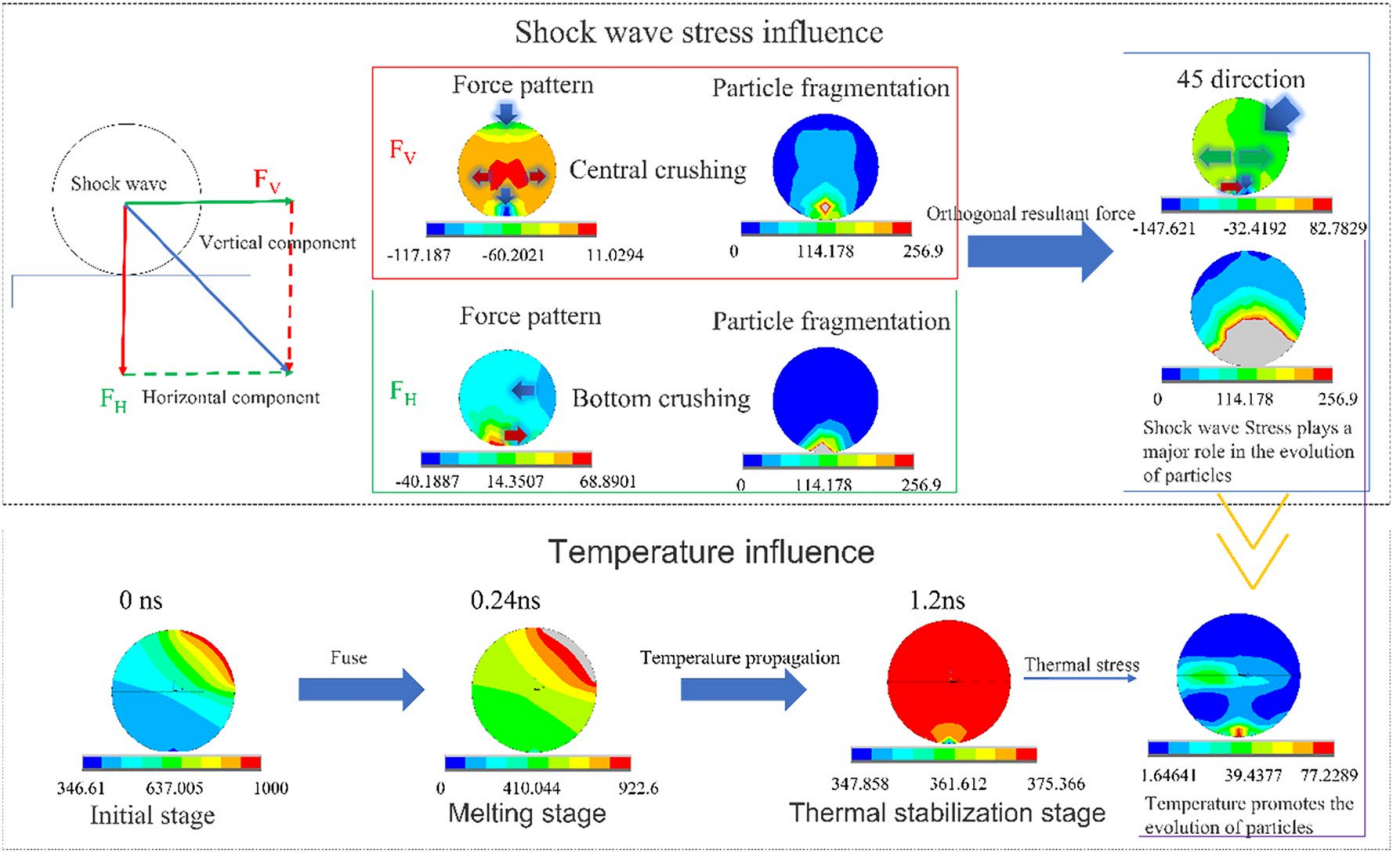
Comparison of the shock wave stress and temperature.

The impact of shock waves on particles can be roughly divided into two categories: the stress and temperature. The stress wave directly acting on the particles is mainly physical, and a high temperature on the shock wave front propagates to the particles, which mainly plays a chemical role. Through simulation analysis, we can develop the following Fig. 8:

First, the impact of the shock wave stress is analyzed. When the stress wave hits the particle at 45°, the acting force can be orthogonally decomposed into vertical and horizontal components. One of the components is simulated and analyzed separately. By observing the stress pattern, when the particles are subjected to the vertical downward force, the stress wave propagates from the contact surface of the particles to the bottom, and after being reflected at the bottom, it collides with the subsequent stress wave. Then, the particles expand and break near the center of the bottom, with the breaking direction occurring from inside to outside. The graph shows that the broken part of the particles at the bottom is inclined to the center, and the particles are currently broken at the center. Regarding the particles subjected to the horizontal direction, the stress pattern shows that the stress is mainly in the contact position between the particles and the substrate, and the friction force is the main force, while the other parts are evenly stressed. Similarly, by observing the particle breakage diagram, the particles are mainly broken at the contact position with the substrate, and the particles are broken at the bottom at this time. Then, the orthogonal resultant force of the two forces is used to obtain the force being applied at 45°. Not only is the bottom of the particle affected by friction, but the center of the particle also tends to expand and break outward. In the particle breaking diagram, the broken area of the particle includes the bottom and the center, and the broken area is significantly larger than the single component.

Next, the influence of the temperature is discussed. At the initial stage, when the wave front reaches the particle surface, the temperature rises rapidly to 1000 K, exceeding the melting point of the particle, and the particle surface melts rapidly. Then, at 0.24 ns, it enters the melting stage, and the surface of the particles is rapidly melted and removed. After the temperature spreads to 1.2 ns, it enters the thermal equilibrium stage, and the temperature of the particles drops rapidly with spreading heat dissipation. At the same time, the temperature spreads rapidly in the particles. However, due to the different thermal conductivities between the aluminum particles and the silicon substrate, the temperature distribution of the other parts is uniform except for the notable temperature difference between the particles and the substrate, which gradually approaches room temperature (298 K). The thermal stress of the particles is simulated by the temperature distribution of the particles, and the thermal stress cloud map is obtained. Moreover, because the temperature is uniformly distributed in the particles and no large stress occurs, a large temperature difference occurs at the junction of the particles and substrate. Thus, the stress, which is 77.2289 MPa, is also significant. However, compared with the yield stress of 121.5 MPa, it cannot affect the disintegration of particles, so the thermal stress has little effect on the phase transformation of the particles.

Compared with the simulation results of the stress and temperature, the breakage of particles is mainly caused by cracking under the action of stress. However, at the beginning of contact, the temperature causes a melting reaction on the surface of particles, and in the subsequent reaction, it mainly accelerates the cracking of the catalytic stress. Therefore, the impact of the shock wave on the particles is mainly caused by the stress wave.

We discussed the situation when the horizontal and vertical components are the same and the force angle is 45°. However, for actual removal, the stress direction of the particles on the substrate is related to the distribution direction of the particles. Moreover, the particles approach the plasma as the stress angle decreases, and vice versa. To determine the force difference of the particles at different force angles, the force surface is changed to forces with different normal angles by modifying the simulation model. After applying the force again, the following simulation results are obtained.

In the figure, the blue line is the average stress of the particles, and the red line is the ratio of the broken area to the total area of particles obtained by setting the breaking threshold. The curve shows that with an increase in the force angle of the particles, the average stress of the particles first expands, reaches a maximum at 45°, and then gradually decreases. The changing trend of the crushing degree is consistent with this trend. Additionally, this result is consistent with the previous results of the separation analysis of horizontal and vertical forces. Furthermore, when the horizontal and vertical components of the particle are the same, the stress is the largest.

**Figure 9:**
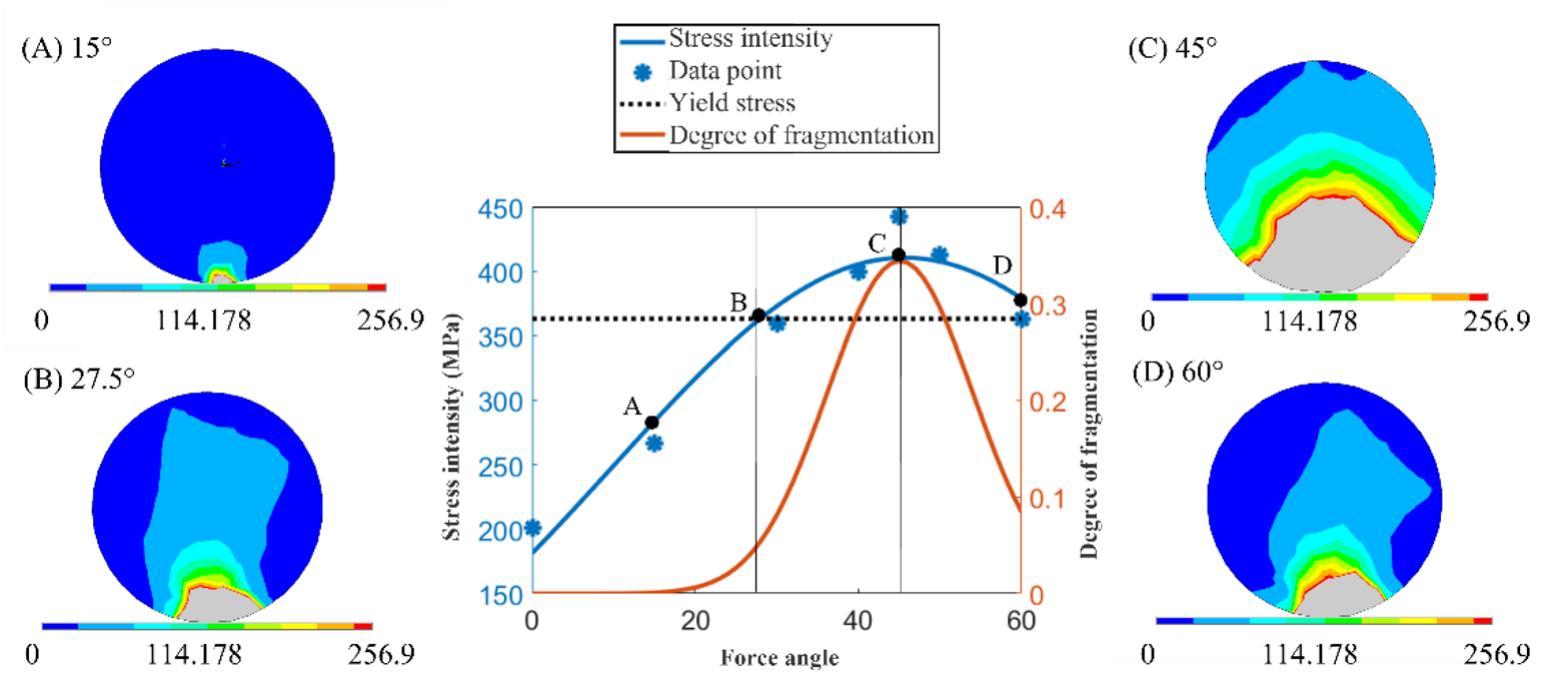
Image of the angle on the particle breakage.

The intersection of the yield stress and the threshold line with the average stress in Fig. 9 is approximately 27.5°. Therefore, when the force angle of the particles is in the range of 0°–27.5°, the average stress value of the particles is lower than the threshold value, and the degree of particle breakage is small. Figure 9A and B represent these concepts. When the force angle of the particles is in the range of 27.5°–45°, the average stress of the particles is higher than the threshold and gradually increases. In addition, the degree of particle breakage also gradually increases until the maximum force is reached at 45°, as shown in Fig. 9B. Afterward, when the force angle of the particles is in the range of 45°–60°, the average stress of the particles decreases again. Thus, the crushing degree gradually begins to decrease, as shown in Fig. 9D.

### Effect of the particle size

**Figure 10:**
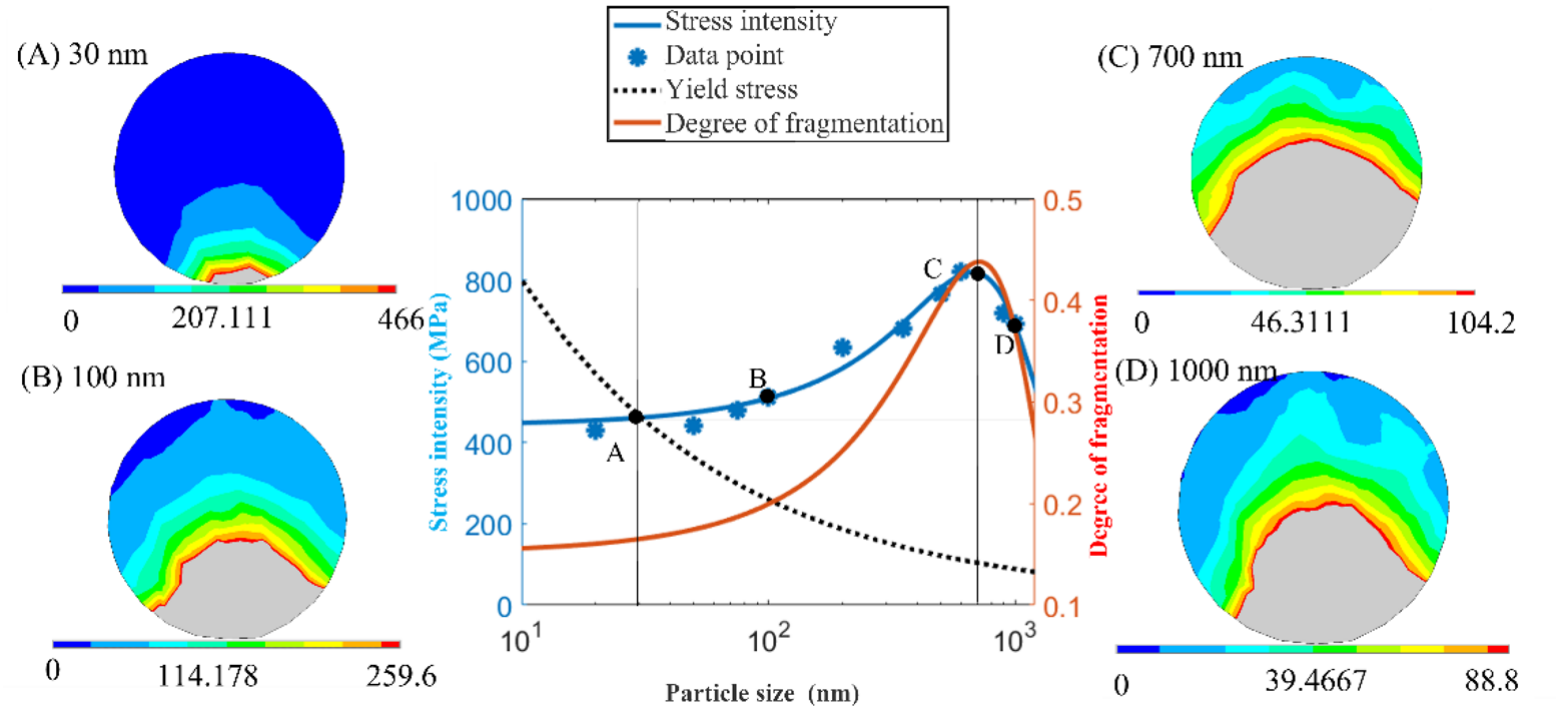
Image of the particle size versus the particle breakage.

First, the influence of the shock wave characteristics on particles is discussed. Then, the influence law of particle size changes on the evolution of particles is discussed. According to the previous model, the influence law can be observed by changing the particle size while maintaining other conditions unchanged and selecting the 45° stress with the most severe crushing degree. Because the influence of the temperature on the particle size is not significant, the change in the stress on different particle sizes is discussed emphatically. The simulation results are shown in Fig. 10.

The blue line in Fig. 10 is a fitting curve of the average stress. With an increase in the particle size, the stress of the particles first increases and then decreases, reaching a maximum at approximately 600 nm. The broken degree curve of the red line is consistent. According to the Hertz contact formula^25^, the following equation can be obtained:

6 $$ a = 0.986\sqrt[3]{{PR\left( {\frac{{1 - u_{1}^{2} }}{{E_{1} }} + \frac{{1 - u_{2}^{2} }}{{E_{2} }}} \right)}} $$

where a is the radius of the contact deformation circle; P is the applied load; R is the radius of the particle; and u_1_, u_2_, E_1,_ and E_2_ are the Poisson’s ratios and Young’s moduli of the two contact materials, respectively. When the particle size is small, the contact area changes little. Therefore, when the particle size is 600 nm, the stress area and the total pressure increase with an increase in the particle size. However, the contact area of the particle hardly changes. Thus, the maximum stress at the bottom of the particle increases correspondingly. When the particle size exceeds 600 nm, the total pressure also increases, but the changing trend of the contact area is stronger.

By adding the yield stress curve to the diagram for comparison, the two curves intersect when the particle size is approximately 30 nm, and the difference reaches a maximum at 700 nm. Therefore, this curve can be divided into three stages.

In the first stage, when the particle size is less than or equal to 30 nm, the particles will not be broken; at this time, the shock wave only has a displacement effect on the particles.

In the second stage, when the particle size is between 30 and 700 nm, the particles are crushed from inside to outside under the action of the shock wave, and the pressure on the particles increases with an increase in the particle size. Until the particle size reaches 700 nm, the difference between the maximum stress and the yield stress is the largest, and the crushing degree is currently the largest.

In the third stage, the difference between the maximum stress and the yield stress of particles larger than 700 nm decreased slightly. However, the decrease was not significant, which indicated that when the particle size increased again, the yield stress of the particles also decreased with the size of the particles although the stress value decreased. Thus, there was little change in the crushing degree of the particles.

### Comprehensive

The basic law of particle evolution is obtained through the separate analysis of the shock wave characteristics and particle size. However, in the experiment, the two variables are interlaced with each other, so a comprehensive discussion on the two variables is needed. Therefore, the stress direction and particle size mentioned above are simultaneously simulated as variables, data are extracted, and the following figure is obtained by fitting the curve.

**Figure 11:**
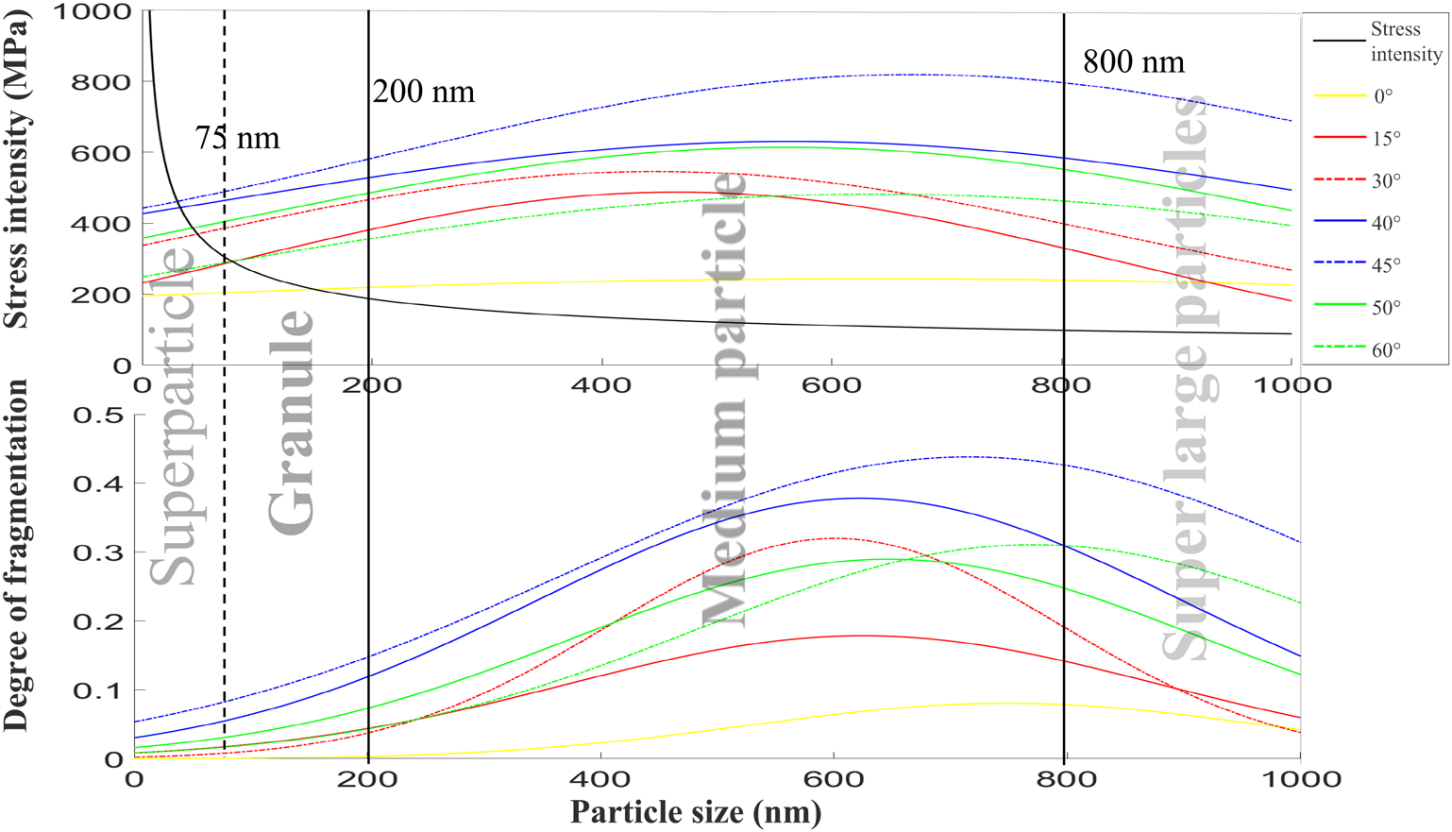
Variation in the average stress of the particles in different regions with the particle size.

Figure 11 shows that with an increase in the particle size, the changing trend of the stress and crushing degree of the particles with different stress angles is the same. This trend represents an increase first and then a decrease. According to the change in the crushing degree, the particle size can be roughly divided into small and large particles, in which the small particles are less than 200 nm. Particles with a particle size less than 75 nm will not be crushed at this time, and they can be regarded as ultra-small particles, which is the limiting particle size for removal. On the other hand, large particles are considered to be larger than 200 nm. They can be divided into medium particles with a particle size in the range of 200–800 nm, and the particle breakage is more significant at this time. Moreover, there is a notable change trend with the change in the particle size. However, particles that are larger than 800 nm can be regarded as super-large particles. Although these particles are significantly broken at this time, the degree of breakage is not considerable, and the change is not notable.

After observing the curves at different angles, the curves can be roughly divided into three regions. The curves in the ranges of 0°–30° and 45°–60° represent the first and third regions, respectively. However, the curve in the range of 30°–45° is higher, and it is the second region that does not intersect with other curves. This is consistent with the change in the particle with the angle, showing that with an increase in the force angle of the particle, the stress and crushing degree of the particle first increases and then decreases. At the same time, it is consistent with the previous division of the experimental phenomena. By observing the curves, the stress of the curves of the first region and the third region is the same when the particles are smaller. However, with an increase in the particle size, the stress of the first region is gradually lower than that of the third region. This is because the third region is subjected to a vertical force component. With an increase in the particle size, the contact area between the third region and the substrate is significantly larger than that of the third region, and the contact area increases, while the flat stress value decreases. The curve of 0° in the first area is significantly lower than all the other curves, and it is almost a horizontal straight line. This is because, in the case of an absolute vertical force, the particles do not show any signs of movement. Therefore, there is no friction force, and the stress value of the particles is less than that of the case with an inclination angle. At the same time, through the Hertz contact formula mentioned earlier, the formula of the maximum stress value of the contact part obtained by dividing the stress by the contact area is as follows^24,25^:

7 $$ f = 0.5784\sqrt[3]{{\frac{p}{{\frac{{1 - u_{1}^{2} }}{{E_{1} }} + \frac{{1 - u_{2}^{2} }}{{E_{2} }}}}}} $$

The maximum stress is not related to the change in the particle size, so the curve in the figure is a horizontal straight line.

Finally, through the above division of the angle and particle size, the combination of the force and crushing degree of the particles is analyzed to obtain the distribution result in the lower part of Fig. 11. With an increase in the force angle or particle size, the degree of particle breakage increases first and then decreases. When the particle size is approximately 500 nm and the force angle is 45°, the crushing degree is maximum, and the removal effect is the best. However, in this case, the residue that is left is also the most. When the force angle of particles is in the range of 45°–60°, large particles and small particles are removed by sliding and rolling, and the damage degree is low. Regarding particles with a force angle in the range of 0°–30°, the crushing degree is not high. Moreover, the removal effect is the worst, and the substrate residue is the most because the particles are subjected to a vertical force.

**Figure 12:**
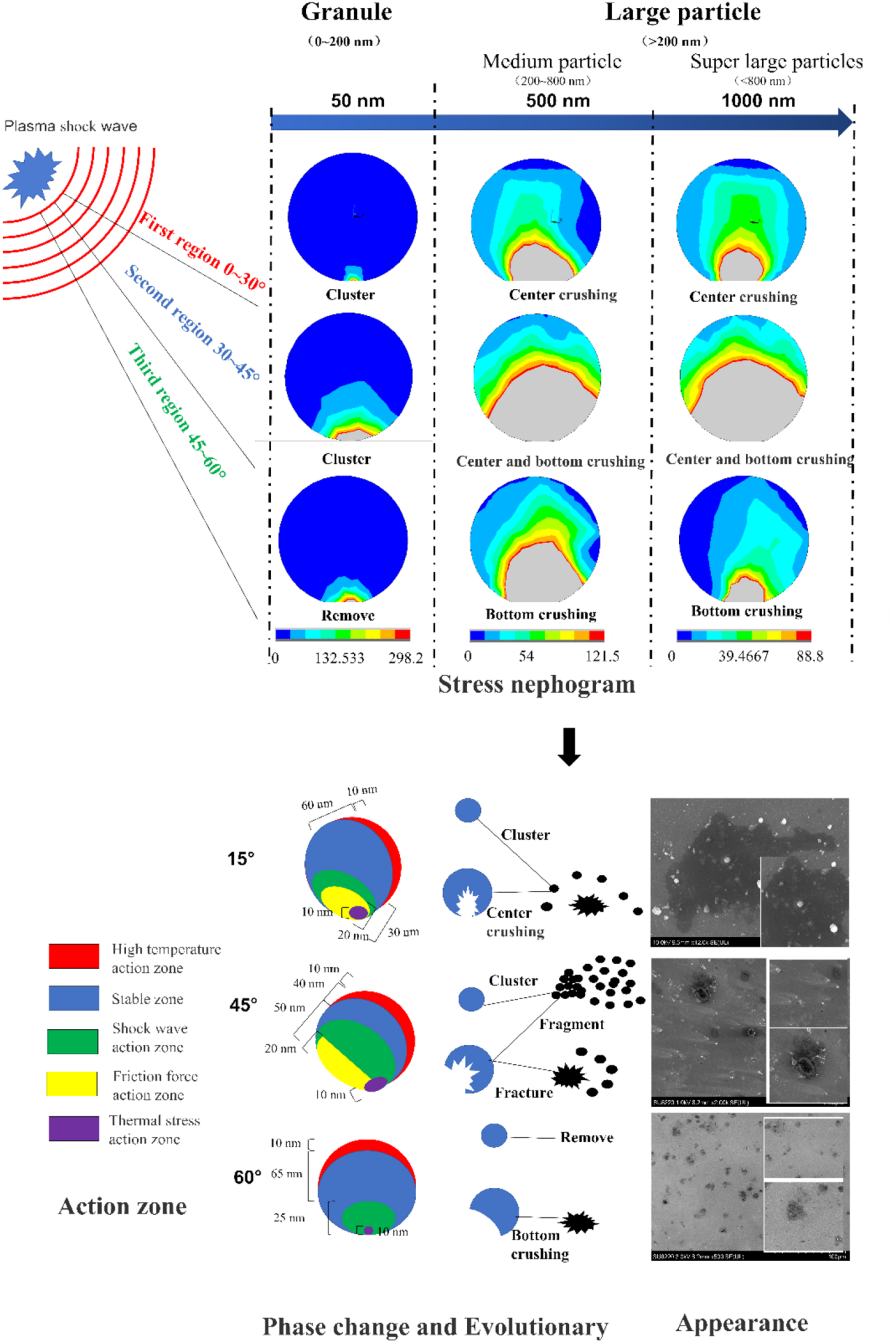
Phase transformation evolution of the particles.

Through the above analysis, the phase change and evolution of the particles can be divided into three regions, which correspond to the previous experimental phenomena, as shown in Fig. 12.

In the first region, ranging from 0° to 30°, when the laser generates the plasma shock wave and touches the particles, the surface temperature of the particles rises rapidly and exceeds the melting point of the particles, resulting in the rapid melting of the contact layer that is approximately 10 nm thick on the surface of the particles. Then, the temperature of the particles dropped rapidly and spread uniformly across the particles. However, at the junction of the particles and substrate approximately 10 nm thick, a certain thermal stress occurred due to the difference in the thermal conductivity between the aluminum particles and the silicon substrate. This thermal stress did not reach the yield stress of the particles, so the particles were not broken. Moreover, the stress was dominated by the vertical force, so the friction force on the particles in the horizontal direction was small. The central area of the particles is squeezed by the stress wave and shock wave reflected by the substrate to form the shock wave action area, which is dominated by central crushing and is approximately 25 nm thick. In this region, the center of the larger particles (particle size larger than 180 nm) is broken and melted under high temperature and pressure, leaving a black spot in the original position. In addition, the broken pieces are scattered around, forming an annular belt around the black spot. Small particles (particle size less than 180 nm) are not broken, and the parts near the large particles converge with the broken clusters of the large particles, which are scattered in the black spots together to form an endless belt. Some of them cluster with each other or bounce on the substrate and then fall back to the substrate, forming a small particle distribution area.

In the second region, ranging from 30° to 45°, the influence of the temperature on the particles is similar to that in the previous region. The upper horizontal stress component is equivalent to the vertical stress component, at which time both the average stress of the particles and the failure area are the largest. The movement of the particles creates a stronger friction force on the contact area between the particles and the substrate so that the area with a thickness of 20 nm at the bottom of the particles is the friction force action area. At the same time, the particles will also be squeezed by the upper and lower shock waves, so that the area that is 30 nm thick upward is the shock wave action area. In this area, larger particles are displaced on the base, and the contact between the bottom and the base is broken under large friction stress, leaving residual fragments at the black spots. At the same time, due to the vertical stress, the center of the particles is broken and melted to some extent, leaving broken residues behind the black spots and forming a removal route. However, under the action of the shock wave, small particles break and melt at the bottom and gather with the fragments and clusters emitted by large particles before forming an extremely unstable aggregate. Thus, a comet head is formed, and it is blown away again under the action of the subsequent shock wave and spreads out behind the comet head, forming a comet tail.

In the third region, ranging from 45° to 60°, the influence of the temperature is similar to that of the previous region, but the horizontal component of the stress gradually increases and the vertical component decreases. Thus, the stress in the center of the particles decreases. Moreover, the particle breakage is only in the bottom region of 30 nm, in which the region with 20 nm thickness is affected by the friction force, and the remaining 10 nm thickness is the active region of the shock wave. In this area, the friction stress of large particles increases, and the bottom is damaged to some extent, leaving a relatively large residue at the black spots. At the same time, no excessive residue occurs around the black spot because the vertical component is less and the particle center is relatively stable. Under the action of a large horizontal component of the stress, the small particles can be relatively removed without leaving too much residue.

## Conclusion

In this study, the influence of the phase transition evolution of micro- and nanoparticles in the process of laser-plasma cleaning and its influence on the cleaning quality were analyzed. It was found that under the action of high pressure and temperature of the laser-plasma shock wave, different action regions of particles appeared, which could be divided into a high-temperature action region, stability region, shock wave action region, friction force action region, and thermal stress action region. These regions caused the non-uniformity of the particle phase transformation characteristics, and the high-temperature region melted, while other areas were broken. These distribution characteristics were affected by the action angle of the shock wave. According to different angles, the cleaning area could be divided into three areas: 0°–30°, 30°–45°, and 45°–60°. Its main feature was that the crushing degree of the particles increased and then decreased with an increase in the stress angle. Otherwise, the particle space phase transition was also affected by its particle size. With an increase in the particle size, the fragmentation degree also increased and then decreased. Through the above conclusions, in practical applications, the force angle could be selected according to the required particle size. For example, the best removal area of large particles (d > 500 nm) was in the range of 30°–45$^\circ$, and the best removal area of small particles (d < 500 nm) was 45°–60°. These results provide reference and guidance for plasma cleaning technology in the future.

## Data Availability

The datasets used and/or analyzed during the current study are available from the corresponding author on reasonable request.
